# Emerging Trichomonad Infections in Companion Animals: Rapid Visual Detection of *Pentatrichomonas hominis* and *Tritrichomonas foetus* Using an RPA‐CRISPR/Cas12a Assay

**DOI:** 10.1155/tbed/9995679

**Published:** 2025-12-12

**Authors:** Yang Zou, Zhi-Wen Yao, Tao Xiao, Ying-Rui Ma, Jun He, Liu-Min Chen, Xiao-Qing Chen

**Affiliations:** ^1^ College of Animal Science and Technology, Jiangxi Agricultural University, Nanchang, 330045, China, jxau.edu.cn; ^2^ State Key Laboratory of Veterinary Etiological Biology, Key Laboratory of Veterinary Parasitology of Gansu Province, Lanzhou Veterinary Research Institute, Chinese Academy of Agricultural Sciences, Lanzhou, 730046, China, caas.cn; ^3^ National Key Laboratory for Swine Genetic Improvement and Production Technology, Ministry of Science and Technology of China, Jiangxi Agricultural University, Nanchang, China, jxau.edu.cn

**Keywords:** companion animal, *Pentatrichomonas hominis*, RPA-CRISPR/Cas12a, *Tritrichomonas foetus*, veterinary diagnostics

## Abstract

*Pentatrichomonas hominis (P. hominis)* and *Tritrichomonas foetus (T. foetus)* are prevalent intestinal protozoa. *P. hominis* is associated with chronic diarrhea in humans and animals, whereas *T. foetus* causes gastrointestinal disease in companion animals and reproductive‐tract infection in cattle. Rapid and accurate identification of these infections at the point‐of‐care (POC) is crucial for the diagnosis and effective management of zoonotic diseases. In this study, we developed two novel recombinase polymerase amplification (RPA) assays coupled with CRISPR/Cas12a detection. The dual‐species assay, using a lateral‐flow format, targeted species‐specific regions of the 18S rRNA gene of *P. hominis* and *T. foetus*, and under ideal conditions, delivered visual results within 40 min for a single sample at 37°C. *P. hominis*‐specific assay: To differentiate *P. hominis* in mixed infections with *T. foetus*, a second assay targeted the highly conserved *Spo11-1* gene of *P. hominis*. Optimal crRNA‐412 and RPA primers were selected for maximal Cas12a cleavage efficiency. Analytical sensitivity and specificity were compared with conventional nested polymerase chain reaction (PCR) and Sanger sequencing. The results showed that The dual‐species assay detected as few as 50 DNA copies/µL of either parasite with no cross‐reactivity to *Giardia lamblia*, *Cystoisospora canis*, *Cryptosporidium* spp., *Toxoplasma gondii*, *Toxocara canis*, and *Toxascaris leonina*. Among 70 fecal samples of companion animal (48 dogs and 22 cats), 14 (29.2%) dogs tested positive for *P. hominis*, and eight cats (36.4%) tested positive for *T. foetus* by nested PCR. Due to financial and logistical constraints, we selected a smaller subset for subsequent analysis with the RPA‐CRISPR/Cas12a lateral‐flow strip (LFS) assay, which showed 100% diagnostic concordance with PCR. The Spo11‐1 assay achieved a limit of detection of 20 DNA copies/µL and specifically recognized *P. hominis* among a panel that included seven non‐target protozoa and helminths. Validation on 10 additional canine and feline samples (four positives and six negatives) showed complete agreement with nested‐PCR results. In conclusion, this CRISPR–based diagnostic approach significantly enhances the efficiency and accuracy of Trichomonads detection, offering a practical, cost‐effective solution particularly suitable for veterinary and potentially human healthcare diagnostics in resource‐limited settings.

## 1. Introduction

*Pentatrichomonas hominis* (*P. hominis*) and *Tritrichomonas foetus* (*T. foetus*) are two agents of organisms that belong to the group known as Trichomonads. They are commonly associated with gastrointestinal disorders in companion animals and have garnered increasing attention due to their zoonotic potential and veterinary importance [1]. *P. hominis* has been found in the gastrointestinal tract of various mammalian hosts, including humans [2], domestic pets [3], livestock [4–7], non‐human primates [8, 9], farmed wild animals, rex rabbits, and fur‐bearing species [10]. Transmission occurs primarily via the fecal‐oral route, and infections have been implicated in gastrointestinal and respiratory illnesses, particularly among vulnerable populations such as children and the elderly [11]. Notably, a previous study reported a *P. hominis* infection rate as high as 41.54% in patients with gastrointestinal malignancies, indicating its potential association with serious health conditions and underscoring its relevance to public health [12]. *T. foetus*, initially recognized as the causative agent of bovine trichomoniasis, was later identified as an emerging enteric pathogen in domestic cats [13, 14]. Cats infected with *T. foetus* possibly present large‐bowel diarrhea characterized by the presence of mucus or fresh blood in the feces, which can result in poor body condition and colonic inflammation [15]. Due to its high transmissibility in multicat environments, such as shelters and catteries, *T. foetus* infection has become an increasing concern in feline medicine.

Current detection methods for trichomonads primarily rely on microscopy, culture‐based techniques, polymerase chain reaction (PCR) assays, and loop‐mediated isothermal amplification (LAMP) [16, 17]. Recently, recombinase polymerase amplification (RPA), an isothermal amplification technique, has been integrated with the CRISPR/Cas12a system, revolutionizing point‐of‐care (POC) biosensing by providing simplicity, speed, and high specificity [18, 19]. For instance, RPA coupled with CRISPR/Cas12a has been successfully used to detect *Tritrichomonas vaginalis* (*T. vaginalis)* in clinical samples [20], highlighting the broad applicability of this approach for trichomonads diagnostics. While PCR is highly sensitive, it can be prone to contamination and requires more sophisticated equipment. Unlike conventional PCR, which requires thermal cyclers and complex protocols, RPA is an isothermal amplification method that does not need temperature cycling. LAMP, on the other hand, is also isothermal and provides fast results, but it often lacks the specificity offered by CRISPR–based detection [21].

In this study, we developed dual‐species and single‐species assays for the highly specific detection of *P. hominis* and *T. foetus* by coupling RPA with CRISPR/Cas12a–based nucleic acid detection and a lateral‐flow strip (LFS) readout. The assays allow for result visualization without the need for complex instrumentation, making it especially well‐suited for field diagnostics and resource‐limited settings. The assays offers high sensitivity and specificity, with no cross‐reactivity to other protozoa. Furthermore, under ideal conditions, the entire workflow, from sample preparation to result visualization, can be completed within 40 min for a single sample, significantly reducing diagnostic time compared to conventional methods. This approach represents a valuable tool for on‐site surveillance and early intervention of protozoan infections in veterinary and potentially zoonotic contexts, and offers a scalable strategy adaptable to other parasitic pathogens.

## 2. Materials and Methods

### 2.1. Sample Preparation

A total of 48 canine fecal samples were collected from a police dog training facility and 22 feline fecal samples were obtained from multiple veterinary clinics located in Nanchang, Jiangxi Province, China, between 2022 and 2024. Approximately 50 g of each fecal sample was collected in sterile sampling tubes. Sample metadata, including host species and collection details, were recorded immediately. Samples were transported to the laboratory under cold chain conditions using foam containers with ice packs. Upon laboratory arrival, aliquots were prepared for microscopic parasitological examination and subsequent DNA extraction. The 200 mg of fecal sediment were used for genomic DNA extraction using the OMEGA E.Z.N.A. Stool DNA Kit, following the manufacturer’s instructions. Proteinase K digestion and silica‐membrane‐based DNA extraction with multiple wash steps were employed to remove PCR inhibitors from the fecal samples. Samples of *P. hominis* and *T. foetus* were isolated during this study. Additionally, genomic DNA from *Giardia lamblia* (*G. lamblia*), *Cryptosporidium* spp., *Toxoplasma gondii* (*T. gondii*), *Cystoisospora canis* (*C. canis*), *Toxocara canis* (*T. canis*), and *Toxascaris leonina* (*T. leonina*) were provided by the Parasitology Laboratory, College of Veterinary Medicine, Shanxi Agricultural University.

### 2.2. DNA Extraction

Approximately 250 mg of each fresh fecal sample from dogs and cats was homogenized and digested with proteinase K. Genomic DNA was subsequently extracted using the TIANamp Genomic DNA Kit (TIANGEN, DP328, Beijing, China), according to the manufacturer’s protocol. Extracted DNA was stored at −80°C until further analysis.

### 2.3. Construction of the pMD19‐T‐18S rRNA Plasmid

A 339‐bp fragment of the 18S rRNA gene, corresponding to a region conserved in both *P. hominis* and *T. foetus*, was amplified by nested PCR. In parallel, a *P. hominis*‐specific fragment of the *Spo11-1* gene was amplified by conventional PCR. *Spo11-1* was selected because of its crucial role in meiotic recombination, a function that is highly conserved in *P. hominis*, and its minimal homology with comparable genes in other related species. Following amplification, the PCR product was purified using a gel extraction procedure. The purified DNA fragment was then ligated into the pMD19‐T vector to construct a recombinant plasmid, designated pMD19‐T‐18S and pMD19‐T‐Spo11‐1 rRNA, serving as a positive control template. The resulting plasmid was transformed into *Escherichia coli* DH5*α* competent cells, and positive clones were confirmed by colony PCR and Sanger sequencing. The confirmed pMD19‐T‐18S and pMD19‐T‐Spo11‐1 rRNA plasmid was extracted using a commercial plasmid miniprep kit, quantified, and subsequently diluted to appropriate concentrations. Aliquots of the plasmid were stored at −20°C for downstream applications, including CRISPR/Cas12a–based detection assays.

### 2.4. Design of Target DNA

The 18S rRNA gene (GenBank: OM763804.1), widely utilized for trichomonad identification, was selected for target region identification. Conserved sequences shared by *P. hominis* and *T. foetus* were identified via sequence alignment. Candidate crRNA target sites were designed downstream of TTTN PAM motifs using an online tool (http://grna.ctegd.uga.edu/). Ten target sequences (PT‐1 to PT‐10) overlapping previously validated amplification regions were chosen (Table S1). To further enhance assay specificity for *P. hominis*, a positive‐control plasmid containing a partial fragment of the *Spo11-1* gene was constructed. Four CRISPR target sites were then selected with the online tool at http://grna.ctegd.uga.edu/ and designated PH‐1 to PH‐4 (Table S2).

### 2.5. crRNA Synthesis and Screening

A universal forward primer (crRNA‐F) was generated based on the structure comprising the T7 promoter (TAATACGACTCACTATAGG), LbCas12a scaffold (aatttctactaagtgtagat), and the target sequence. Ten reverse primers (crRNA‐R, PT‐1–PT‐10) specific to the 18S rRNA targets (Table 1) and four additional reverse primers (PH‐1toPH‐4) specific to the *Spo11-1* targets of *P. hominis* were designed individually and are listed in Table 2. All primers were synthesized by Sangon Biotech (Shanghai, China). Each crRNA template was generated by PCR using previously validated genomic DNA from *P. hominis* or *T. foetus* as the template. The PCR mixture (25 μL) consisted of 1 μL each forward and reverse primer (10 μM), 12.5 μL Premix Taq (Ex Taq Version 2.0 plus dye), and ddH_2_O. Cycling parameters were 95°C initial denaturation (5 min); 35 cycles of 95°C (30 s), 60°C (45 s), and 72°C (10 s); final extension at 72°C (10 min), and 12°C hold (5 min). PCR products were confirmed by 2% agarose gel electrophoresis.

**Table 1:** crRNA primers designed for the dual‐species RPA‐CRISPR/Cas12a‐LFS.

Primer’s name	Primer’s sequence
crRNA‐F	GAAATTAATACGACTCACTATAGGGTGGATAATTTCTACTGTTGTAGAT
crRNA‐175‐R	TAAGTTTCCCCGTGTTGATTATCTACAACAGTAGAAATTATCCACCCTATA
crRNA‐215‐R	GACCCGAAGCCTGTCAGTCAATCTACAACAGTAGAAATTATCCACCCTATA
crRNA‐239‐R	ACCACCAAAAGCAATATCCTATCTACAACAGTAGAAATTATCCACCCTATA
crRNA‐254‐R	CACCAACGGCCATGCACCACATCTACAACAGTAGAAATTATCCACCCTATA
crRNA‐148‐R	TTAATTTGAATCAACACGGGATCTACAACAGTAGAAATTATCCACCCTATA
crRNA‐78‐R	GGAACTACGACCGCAAGGCTATCTACAACAGTAGAAATTATCCACCCTATA
crRNA‐69‐R	CCAAGAACTATGATTTCTCTATCTACAACAGTAGAAATTATCCACCCTATA
crRNA‐70‐R	CCCAAGAACTATGATTTCTCATCTACAACAGTAGAAATTATCCACCCTATA
crRNA‐212‐R	CCGAAGCCTGTCAGTCATAAATCTACAACAGTAGAAATTATCCACCCTATA
crRNA‐213‐R	CCCGAAGCCTGTCAGTCATAATCTACAACAGTAGAAATTATCCACCCTATA

**Table 2:** crRNA primers designed for the *P. hominis*‐specific RPA–CRISPR/Cas12a‐LFS assay.

Primer’s name	Primer’s sequence
crRNA‐412	GGGUGGAUAAUUUCUACUGUUGUAGAUAACCAACACUAUUUGGUUCU
crRNA‐565	GGGUGGAUAAUUUCUACUGUUGUAGAUGCUCGUCCACCACUAUCUGC
crRNA‐356	GGGUGGAUAAUUUCUACUGUUGUAGAUAACAACUGCUAGUACUUUGA
crRNA‐566	GGGUGGAUAAUUUCUACUGUUGUAGAUGGCUCGUCCACCACUAUCUG

### 2.6. RPA Primer Design and Selection

RPA primers were designed in Primer Premier 6.0 according to standard RPA guidelines. For the dual‐species assay, eight forward and 10 reverse primers targeting *P. hominis* and *T. foetus* were generated, whereas the *P. hominis*‐specific assay employed eight forward and six reverse primers. All oligonucleotides were synthesized by Sangon Biotech (Shanghai, China) (Tables S3 and S4).

### 2.7. RPA‐CRISPR/Cas12a Assay Establishment

Amplification products from RPA reactions required purification prior to CRISPR–Cas12a detection to remove inhibitory components. Purification utilized a commercial DNA purification kit following manufacturer guidelines. The RPA reaction utilized the TwistDx Liquid Basic kit, assembled according to manufacturer instructions with the selected primer pairs. Purified amplicons were analyzed using fluorescence and lateral flow assays. For fluorescence detection, 2 μL purified RPA product was mixed with 5 μL Cas12a enzyme, 3 μL 10 × NEBuffer r2.1, 1 μL fluorophore‐quencher probe (YgCas12a), 1 μL crRNA, and nuclease‐free water to 30 μL total volume, incubated at 37°C for 40 min using an Easy PGX real‐time PCR system. FAM fluorescence signals were recorded every minute. For lateral LFS detection, 10 μL purified RPA product was mixed with 5 μL Cas12a enzyme, 3 μL 10 × NEBuffer r2.1, 1 μL LFS probe (TzCas12a), 1 μL crRNA, and nuclease‐free water to 30 μL total volume, incubated at 37°C for 30 min. The volume was adjusted to 50 μL and an LFS was inserted for 5 min before visual interpretation.

### 2.8. RPA‐CRISPR/Cas12 Combined Lateral Flow Chromatography Test Strip

The CRISPR/Cas12a–based LFS used in this study is coated with streptavidin (SA) at the C‐line and anti‐mouse secondary antibody at the T‐line. Colloidal gold‐labeled anti‐FITC/FAM monoclonal antibodies are employed for detection. The TzCas12a probe, synthesized with biotin at one end and FAM or FITC at the other, ensures colloidal gold is captured at the C‐line. Upon cleavage by Cas12a, the gold bound to the cleaved fragment is no longer captured at the C‐line and migrates upward, forming a signal at the T‐line. During probe concentration optimization, the TzCas12a probe binds colloidal gold in the conjugate pad, and as the solution moves upward, the probe is adsorbed at the C‐line. When the probe concentration is lower than the gold, unbound gold‐labeled antibodies migrate further and are captured at the T‐line.

### 2.9. Analytical Sensitivity and Specificity

Specificity was assessed by testing genomic DNA from non‐target protozoa and other helminths, including *P. hominis*, *T. foetus*, *G. lamblia*, *Cryptosporidium* spp., *C. canis*, *T. canis*, *T. leonina*, and *T. gondii*. Analytical sensitivity was evaluated by testing serial dilutions (10^0^–10^2^copies/μL) of pMD19‐T‐18S rRNA and pMD19‐T‐Spo11‐1 plasmid DNA. Nuclease‐free water was used as a negative control.

### 2.10. Clinical Sample Detection

Nested PCR was first applied to 70 clinical fecal samples—48 canine and 22 feline—targeting the conserved 18S rRNA region of *P. hominis* and the ITS1‐5.8S‐ITS2 region of *T. foetus*. Amplicons were sequenced by Sangon Biotech (Shanghai, China) and their identities confirmed by BLAST analysis against the NCBI database. Eight PCR‐positive samples (three canine and five feline) and 17 PCR‐negative samples were then examined with the dual‐species RPA‐CRISPR/Cas12a lateral‐flow assay developed in this study. Finally, four *P. hominis*‐positive and six negative samples were analyzed with the single‐species *P. hominis* RPA‐CRISPR/Cas12a assay.

## 3. Results

### 3.1. Selection of High‐Efficiency crRNAs for Detection of *P. hominis* and *T. foetus*

For the dual‐species RPA‐CRISPR/Cas12a detection assay, crRNAs targeting conserved regions of the *P. hominis* and *T. foetus* 18S rRNA genes were generated via PCR amplification followed by in vitro transcription. A universal forward primer (crRNA‐F) containing a T7 promoter sequence at the 5’ end and 10 distinct reverse primers specific to each target region were used to amplify double‐stranded DNA templates (~47 bp) encoding the crRNAs. These PCR amplicons served as templates for in vitro transcription using T7 RNA polymerase and yielding 47‐bp crRNA transcripts (Figure 1A). The resulting crRNAs‐designated crRNA‐175, crRNA‐215, crRNA‐239, crRNA‐254, crRNA‐148, crRNA‐78, crRNA‐69, crRNA‐70, crRNA‐212, and crRNA‐213 were tested for performance in the CRISPR/Cas12a–based fluorescence detection system. Four of the 10 crRNAs (crRNA‐69, crRNA‐70, crRNA‐212, and crRNA‐213) exhibited robust target binding and efficient Cas12a‐mediated collateral cleavage of a single‐stranded DNA (ssDNA) fluorescent reporter, resulting in strong fluorescence upon target recognition. Among these, crRNA‐70 reached the predefined fluorescence threshold in the shortest time (Figure 1B). Based on these results, crRNA‐70 was selected for further testing.

Figure 1The crRNA screening and RPA condition optimization for sensitive detection for dual‐species RPA‐CRISPR/Cas12a‐lateral flow assay (LFS). (A) The electrophoresis of crRNA by transcription in vitro. Lanes: 1–10: Template for crRNA transcription. (B) The screening of crRNA by fluorescence value. (C) The electrophoresis of F7/R9 RPA primer pair amplification. (D) Electrophoretic analysis of RPA at various temperatures. (E) Electrophoretic analysis of recombinase polymerase amplification at different time intervals. M, DNA maker; NC, Naive control.

**Figure:**
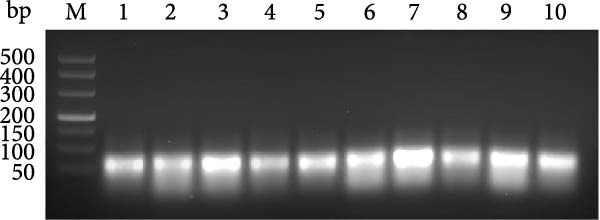
(A)

**Figure:**
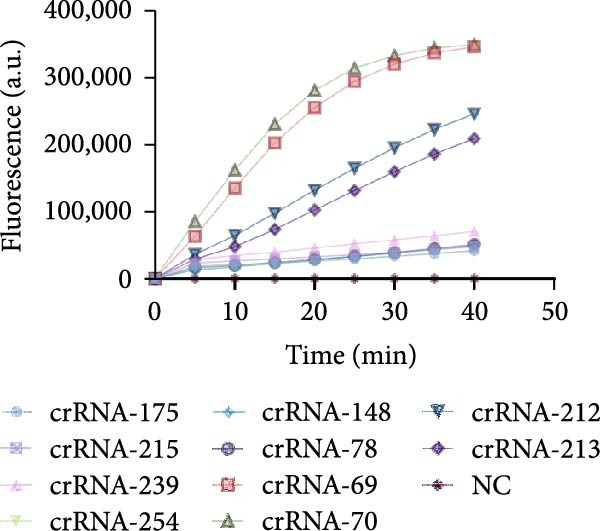
(B)

**Figure:**
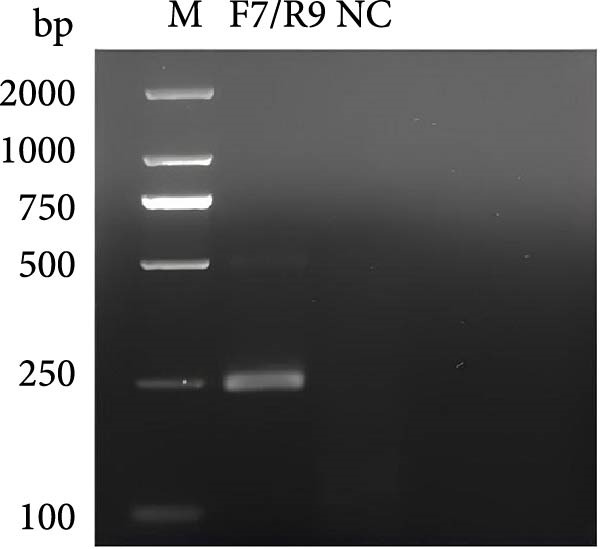
(C)

**Figure:**
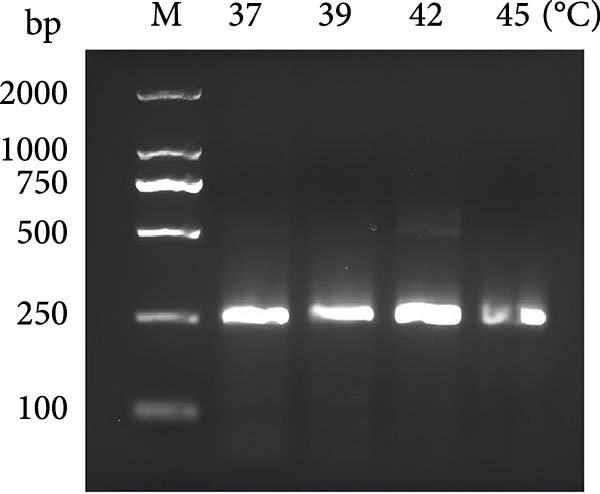
(D)

**Figure:**
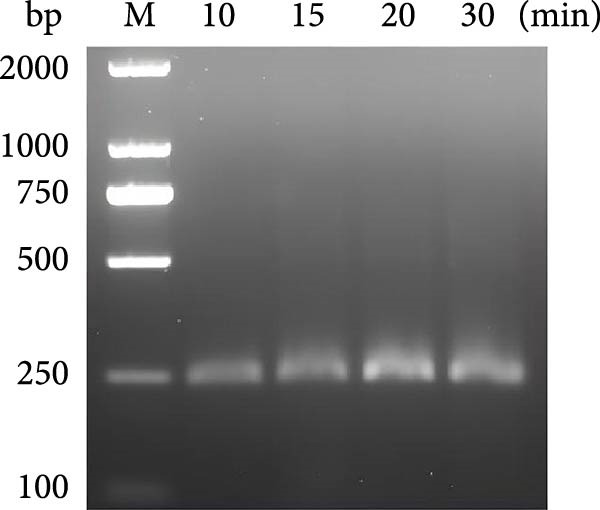
(E)

### 3.2. Establishing an Optimized RPA Protocol for 18S rRNA Detection

The combined RPA‐CRISPR/Cas12a assay for detecting *P. hominis* and *T. foetus* was established by designing RPA primers RPA‐F7 and RPA‐R9 to target the 18S rRNA gene region in conjunction with crRNA‐70 (Table S3 and Figure S1A,B). RPA amplification was carried out using 10^3^ copies of the pMD19‐T‐18S rRNA plasmid as a positive control and nuclease‐free water as a negative control. Agarose gel electrophoresis revealed a distinct band at the expected size for the positive control, while no amplification was observed in the negative control (Figure 1C). To optimize the assay, sensitivity was assessed under varying reaction temperatures and incubation times. The results demonstrated that a temperature of 42°C for 20 min provided the most efficient and distinct amplification signals (Figure 1D,E).

### 3.3. RPA‐CRISPR/Cas12a Assay System Coupled With LFS for Detection

The RPA‐CRISPR/Cas12a–based LFS (RPA‐CRISPR/Cas12a‐LFS) successfully detected target sequences (Figure 2A). In the absence of cleavage, colloidal gold was captured at the C‐line. Upon Cas12a‐mediated cleavage of the TzCas12a probe, colloidal gold failed to be captured at the C‐line and migrated upwards to form a distinct signal at the T‐line. Optimization of the probe concentration showed that as the probe concentration decreased from 50 nM to 1 μM, the T‐line signal intensity gradually diminished. At a probe concentration of 100 nM, the T‐line became undetectable (Figure 2B).

Figure 2Analytical and diagnostic validation of the dual‐species RPA‐CRISPR/Cas12a‐LFS detection assay for *P. hominis* and *T. foetus*. (A) Schematic illustration of the detection of *P. hominis* and *T. foetus* in canine and feline samples based on dual‐species RPA‐CRISPR/Cas12a‐LFS. (B) Screening for optimal probe concentration on lateral flow strips. (C) The sensitivity analysis of dual‐species RPA‐CRISPR/Cas12a‐LFS. (D) The specificity analysis of dual‐species RPA‐CRISPR/Cas12a‐LFS. (E) Diagnostic validation of the RPA‐CRISPR/Cas12a‐LFS for the detection of *P. hominis* and *T. foetus* in canine and feline fecal samples. NC, negative control. 1–17, negative samples. 18–25, positive samples.

**Figure:**
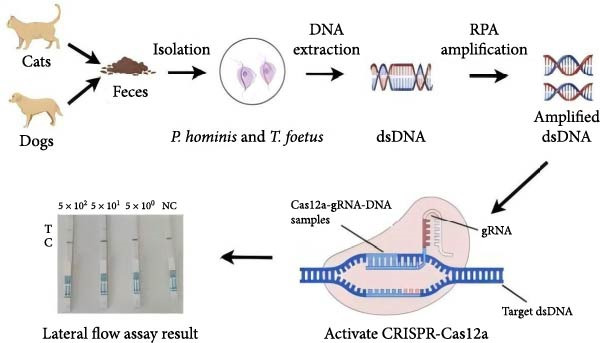
(A)

**Figure:**
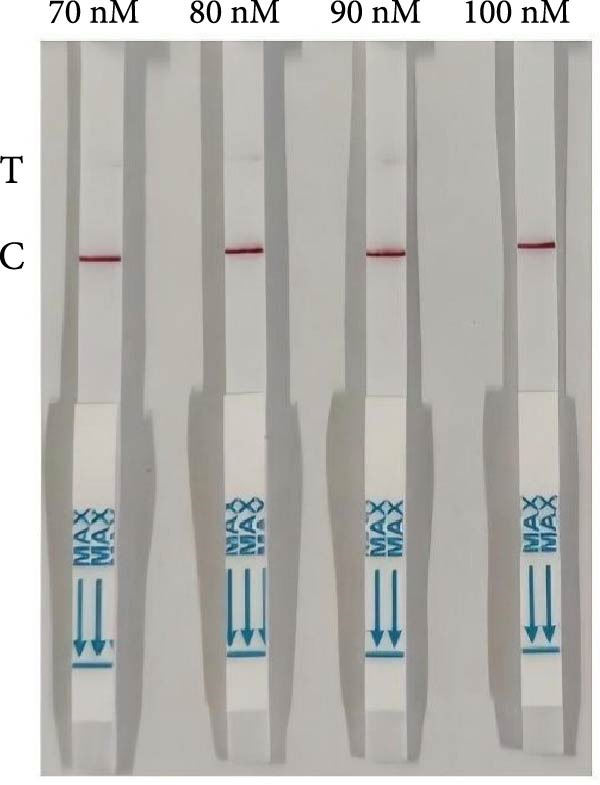
(B)

**Figure:**
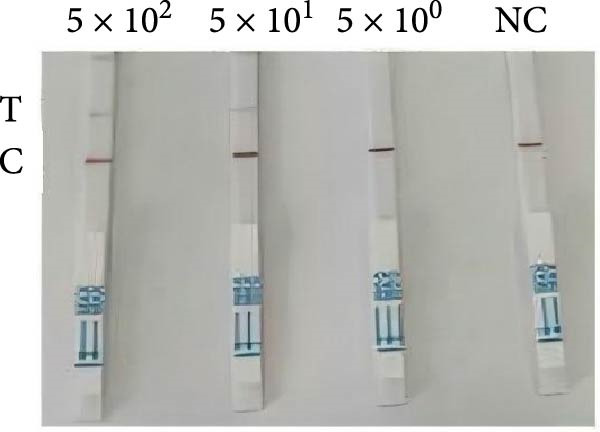
(C)

**Figure:**
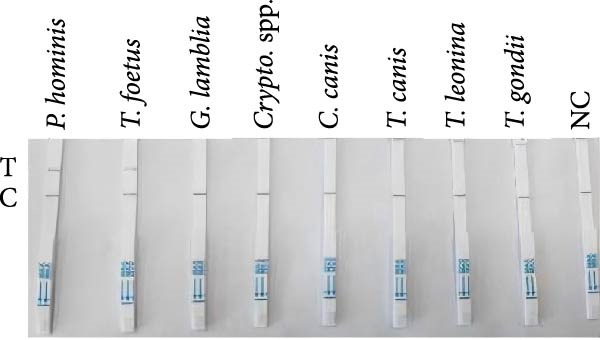
(D)

**Figure:**
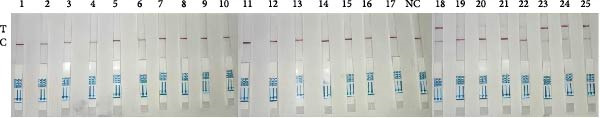
(E)

### 3.4. Assessing the Specificity and Sensitivity of RPA‐CRISPR/Cas12a‐LFS

The specificity of the RPA‐CRISPR/Cas12a detection method was evaluated using genomic DNA samples from *G. lamblia*, *Cryptosporidium* spp., *C. canis*, *T. canis*, *T. leonina*, and *T. gondii* as negative controls, compared with *P. hominis* and *T. foetus* genomic DNA samples. The results showed that only *P. hominis* and *T. foetus* exhibited a band at the T‐line, while the DNA samples from the other six protozoan species showed bands at the C‐line with no signal at the T‐line (Figure 2C). These findings confirm the high specificity of the RPA‐CRISPR/Cas12a detection method. For further sensitivity testing, RPA amplification was performed using positive DNA templates with copy numbers ranging from 5 × 10^0^ to 5 × 10^2^ copies/μL. The results indicated that the minimum detection limit was 5 × 10^1^ copies/μL, as a visible T‐line was observed at this concentration and higher (Figure 2D).

### 3.5. Evaluation of Diagnostic Performance Using RPA‐CRISPR/Cas12a‐LFS

A total of 70 fecal samples, including 48 from dogs and 22 from cats, were collected. Among them, 14 (29.2%) *P. hominis*‐positive samples from dogs and 8 (36.4%) *T. foetus*‐positive samples from cats were successfully sequenced by nested PCR (Figure S2A–C). A total of 25 samples, including eight positive and 17 negative samples, were selected to evaluate the diagnostic performance of the established RPA‐CRISPR/Cas12a‐LFS detection method. The results showed that for the eight positive samples, both the C‐line and T‐line on the test strips displayed visible signals. In contrast, for the 17 negative samples, only the C‐line exhibited a visible signal, while the T‐line remained uncolored (Figure 2E).

### 3.6. Construction of *P. hominis*‐Specific RPA‐CRISPR/Cas12a‐LFS

For the *P. hominis*‐specific RPA–CRISPR/Cas12a assay, four candidate crRNAs (crRNA‐412, ‐565, ‐356, and ‐566) were designed against conserved regions of the *Spo11-1* gene and synthesized commercially. Cleavage kinetics, monitored on an Easy PGX real‐time PCR platform with nuclease‐free water as the no‐template control, showed that crRNA‐412 generated the highest fluorescence signal and was, therefore, selected for further work (Figure 3A). Primer screening identified the RPA pair F1/R6 as optimal for amplifying Spo11‐1 in conjunction with crRNA‐412 (Supporting Information: Table S4). Using 10^3^ copies of the pMD19‐T‐Spo11‐1 plasmid as a positive control, RPA yielded the expected amplicon, confirmed by 2% agarose electrophoresis (Figure 3B).

Figure 3Specific detection of *P. hominis* using a *Spo11-1*‐based RPA‐CRISPR/Cas12a‐lateral flow assay (LFS). (A) The screening of candidate crRNA by fluorescence value for the *P. hominis*‐specific RPA–CRISPR/Cas12a assay. (B) Agarose gel electrophoresis of amplification products generated using the RPA primer pair F1/R6. (C) The specificity analysis of *P. hominis*‐specific RPA–CRISPR/Cas12a‐LFS assay against non‐target parasite. (D) Sensitivity analysis of the assay using serial dilutions of pMD19‐T‐Spo11‐1 plasmid DNA. (E) Diagnostic validation of the *P. hominis*‐specific RPA‐CRISPR/Cas12a‐LFS assay in canine and feline fecal samples. Lanes 1–6, negative samples. NC, negative control. Lanes 7–10, positive samples.

**Figure:**
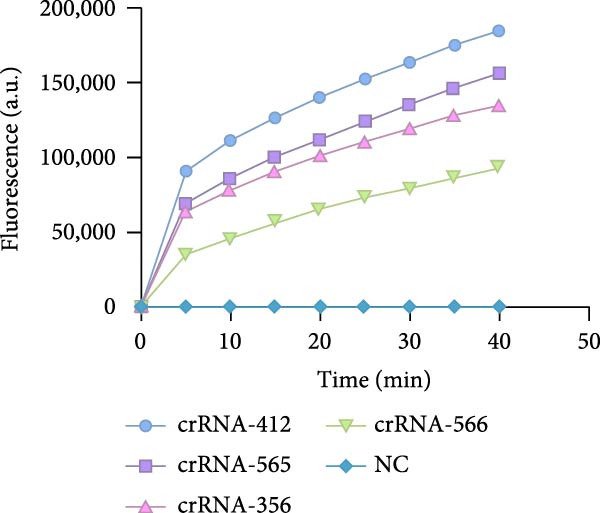
(A)

**Figure:**
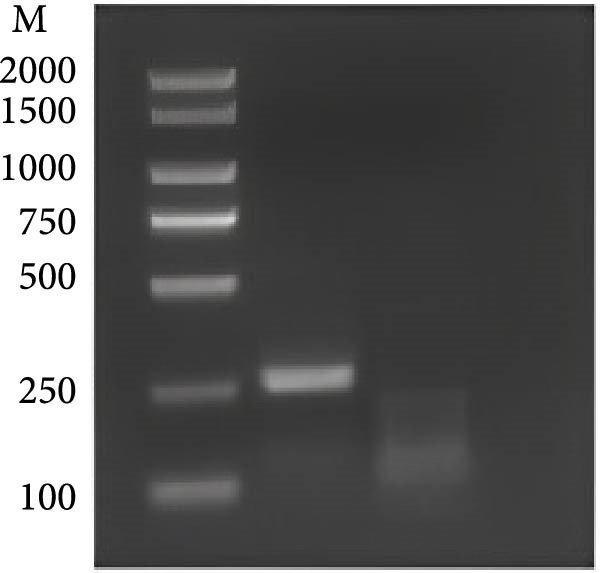
(B)

**Figure:**
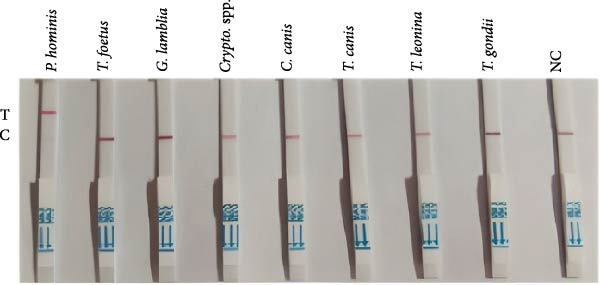
(C)

**Figure:**
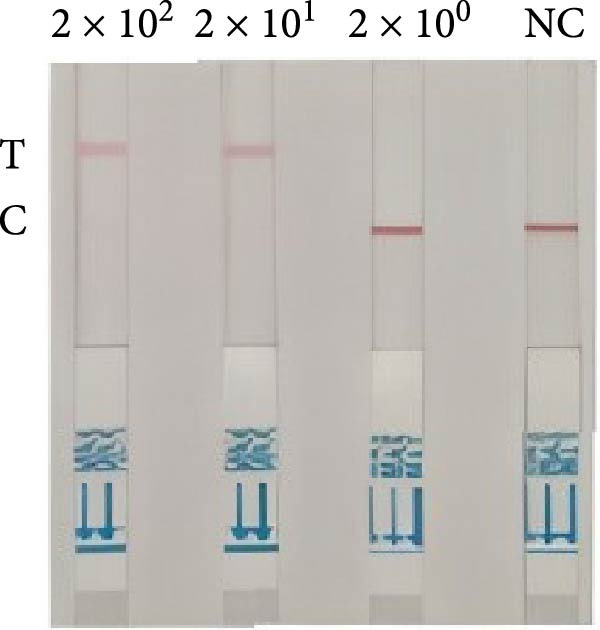
(D)

**Figure:**
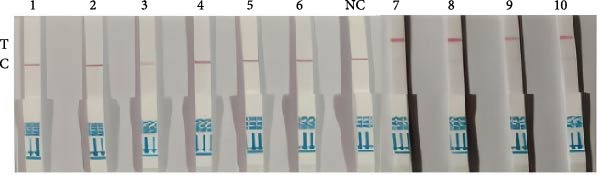
(E)

Specificity testing demonstrated that only *P. hominis* genomic DNA produced a T‐line signal on the LFS, whereas DNA from seven heterologous protozoan parasites generated a C‐line signal only (Figure 3C). Sensitivity analysis revealed a clearly discernible T‐line at plasmid concentrations as low as 2 × 10^1^ copies/µL, establishing a limit of detection of 2 × 10^1^ copies/µL (Figure 3D). Clinical validation with 10 fecal specimens showed 100% concordance with nested‐PCR results: all four PCR‐positive samples yielded dual C‐ and T‐line signals, whereas all six PCR‐negative samples displayed a C‐line only (Figure 3E).

## 4. Discussion

*P. hominis* and *T. foetus* are recognized as significant pathogens affecting companion animal health, particularly responsible for chronic diarrheal conditions in felines, especially in kittens and immunocompromised individuals [1, 16, 22]. Sporadic human infections underscore their zoonotic potential and public health relevance [23]. Current diagnostic approaches for trichomonad infections mainly include direct fecal microscopy, culture techniques, histopathological examination, and PCR assays [24]. Nonetheless, these conventional methodologies are often labor‐intensive, technically demanding, and time‐consuming, limiting their practicality for rapid, on‐site diagnostics or application in resource‐constrained environments. Our diagnostic assay is more cost‐effective than conventional PCR, as it eliminates the need for expensive reagents and complex equipment, such as thermal cyclers and fluorescence readers. By utilizing LFS, the assay is also well‐suited for high‐throughput diagnostics. In contrast to PCR, which requires specialized infrastructure, our assay can be conducted with minimal equipment, including a portable incubator and LFS, thereby reducing both setup and operational costs. The assay provides rapid results and does not depend on extensive laboratory facilities, making it an ideal solution for POC diagnostics, particularly in remote or resource‐limited settings, such as veterinary clinics.

In response to these challenges, the present study developed dual‐species or *P. hominis*‐specific RPA–CRISPR/Cas12a assay designed for the rapid and specific detection of *P. hominis* and *T. foetus*. The dual‐species RPA–CRISPR/Cas12a assay demonstrated high specificity, accurately identifying the target *P. hominis* and *T. foetus* without cross‐reactivity with other protozoan species previously reported [20, 25–27], and exhibited high sensitivity with a detection limit as low as 5 × 10^1^ copies/μL. The diagnostic performance of the assay was evaluated using clinical fecal samples from dogs and cats, and the results were consistent with those obtained from traditional nested PCR, indicating comparable accuracy. For targeted identification of *P. hominis* in mixed infections, a single‐species assay based on the Spo11‐1 locus further lowered the detection threshold to 2 × 10^1^ copies/µL.

Moreover, this novel RPA‐CRISPR/Cas12a assay presents several advantages. Under ideal conditions, it significantly reduces detection time, providing results within approximately 40 min for a single sample, and eliminates the need for complex laboratory equipment. The straightforward visual readout further enhances its practicality for clinical application and particularly in resource‐limited settings.

However, the overall workflow‐including DNA extraction, RPA amplification, post‐RPA purification, and CRISPR–based detection‐involves multiple steps that require hands‐on time and introduces additional opportunities for contamination. While the assay is designed to be rapid, the total time for processing a single sample may exceed 1 h when considering all steps. Furthermore, processing multiple samples will increase the turnaround time, potentially approaching several hours, particularly when factoring in sample preparation and postamplification steps. Therefore, although the method provides rapid results for a small number of samples, the overall turnaround time for larger batches may be longer, particularly in settings where throughput is a crucial. Additionaly, the reliance on proprietary RPA kits and custom LFS limits accessibility, particularly in lower‐resource settings. Further are necessary to rigorously assess the performance of this method across a broader range of clinical samples, especially those with low parasite burdens, to ensure its reliability and robustness under diverse clinical conditions.

## 5. Conclusion

In this study, we have established an RPA‐CRISPR/Cas12a detection assay that marks a significant advancement in the molecular diagnosis of *P. hominis* and *T. foetus* infections. Given its high sensitivity, specificity, and rapid detection time, this assay shows strong potential as a preferred diagnostic tool for trichomoniasis in dogs and cats, especially in resource‐limited settings. This indicates the possibility of using a rapid test for the diagnosis of trichomonads in the future. However, further additional studies are needed, and especially in connection with the clinical condition of the patient.

## Ethics Statement

The animal experiments performed in this study were approved by the Animal Ethics Committee of the Department of Veterinary Preventive Medicine, College of Animal Science and Technology, Jiangxi Agricultural University (Number JXAULL‐2022‐01‐21). All procedures complied with international guidelines for the care and use of laboratory animals.

## Disclosure

All authors proofread and approved the final version of manuscript.

## Conflicts of Interest

The authors declare no conflicts of interest.

## Author Contributions

Xiao‐Qing Chen designed the project. Zhi‐Wen Yao performed the experiments. Zhi‐Wen Yao and Yang Zou analyzed the data. Tao Xiao, Ying‐Rui Ma, Jun He, and Liu‐Min Chen contributed reagents/materials/analysis tools. Yang Zou wrote the manuscript. The manuscript was written through contributions of all authors. Yang Zou and Zhi‐Wen Yao contributed equally to this work.

## Funding

This work was supported by the fund of the National Natural Science Foundation of China (Grant 32202839) and the Jiangxi Province Natural Science Foundation (Grant 20242BAB20312).

## Supporting Information

Additional supporting information can be found online in the Supporting Information section.

## Supporting information


**Supporting Information** Figure S1: The electrophoresis of RPA primer screen. A. The electrophoresis of RPA upstream primer screen; B. The electrophoresis of RPA downstream primer screen. M, DNA Maker DL2000. Figure S2: Electrophoretic analysis of nested PCR amplification for the detection of *P. hominis* and *T. foetus* in fecal samples from dogs and cats A. The nested PCR amplification recognizing of *P. hominis* in canine fecal samples; B. The nested PCR amplification recognizing of *P. hominis* in feline fecal samples. C. The nested PCR amplification recognizing of *T. foetus* in feline fecal samples. M, DNA maker DL2000; NC, Negative control; numbers, canine and feline fecal samples. Table S1: Target sequence designed for 18S rRNA. Table S2: crRNA sequences for detection of *P. hominis* and *T. foetus*. Table S3: RPA primers designed for the detection of *P. hominis* and *T. foetus*. Table S4: RPA primers for amplifying *Spo11-1* gene in conjunction with crRNA‐412.

## Data Availability

The data supporting the findings of this study have not been deposited in a public repository but are available from the corresponding authors upon reasonable request.
