# Supplementary material for: Emerging Trichomonad Infections in Companion Animals: Rapid Visual Detection of Pentatrichomonas hominis and Tritrichomonas foetus Using an RPA‐CRISPR/Cas12a Assay
Source: Transbound Emerg Dis. 2025 Dec 12;2025:9995679. doi: 10.1155/tbed/9995679 (PMC12701322; doi:10.1155/tbed/9995679)
Supplement: Supplementary file 1 — Supporting Information Figure S1: The electrophoresis of RPA primer screen. A. The electrophoresis of RPA upstream primer screen; B. The electrophoresis of RPA downstream primer screen. M, DNA Maker DL2000. Figure S2: Electrophoretic analysis of nested PCR amplification for the detection of P. hominis and T. foetus in fecal samples from dogs and cats A. The nested PCR amplification recognizing of P. hominis in canine fecal samples; B. The nested PCR amplification recognizing of P. hominis in feline fecal samples. C. The nested PCR amplification recognizing of T. foetus in feline fecal samples. M, DNA maker DL2000; NC, Negative control; numbers, canine and feline fecal samples. Table S1: Target sequence designed for 18S rRNA. Table S2: crRNA sequences for detection of P. hominis and T. foetus. Table S3: RPA primers designed for the detection of P. hominis and T. foetus. Table S4: RPA primers for amplifying Spo11-1 gene in conjunction with crRNA‐412. [file TBED-2025-9995679-s001.docx]

**Table S1** Target sequence designed for 18S rRNA

| Name | Sequence（5’-3’） |
| --- | --- |
| PT-1 | AATCAACACGGGGAAACTTA |
| PT-2 | TGACTGACAGGCTTCGGGTC |
| PT-3 | AGGATATTGCTTTTGGTGGT |
| PT-4 | GTGGTGCATGGCCGTTGGTG |
| PT-5 | CCCGTGTTGATTCAAATTAA |
| PT-6 | AGCCTTGCGGTCGTAGTTCC |
| PT-7 | AGAGAAATCATAGTTCTTGG |
| PT-8 | GAGAAATCATAGTTCTTGGG |
| PT-9 | TTATGACTGACAGGCTTCGG |
| PT-10 | TATGACTGACAGGCTTCGGG |

**Table S2** crRNA sequences for detection of *P. hominis* and *T. foetus.*

| **crRNA Name** | **Sequence** |
| --- | --- |
| crRNA-175 | GGGUGGAUAAUUUCUACUGUUGUAGAUUAAGUUUCCCCGUGUUGAUU |
| crRNA-215 | GGGUGGAUAAUUUCUACUGUUGUAGAUGACCCGAAGCCUGUCAGUCA |
| crRNA-239 | GGGUGGAUAAUUUCUACUGUUGUAGAUACCACCAAAAGCAAUAUCCU |
| crRNA-254 | GGGUGGAUAAUUUCUACUGUUGUAGAUCACCAACGGCCAUGCACCAC |
| crRNA-148 | GGGUGGAUAAUUUCUACUGUUGUAGAUUUAAUUUGAAUCAACACGGG |
| crRNA-78 | GGGUGGAUAAUUUCUACUGUUGUAGAUGGAACUACGACCGCAAGGCU |
| crRNA-69 | GGGUGGAUAAUUUCUACUGUUGUAGAUCCAAGAACUAUGAUUUCUCU |
| crRNA-70 | GGGUGGAUAAUUUCUACUGUUGUAGAUCCCAAGAACUAUGAUUUCUC |
| crRNA-212 | GGGUGGAUAAUUUCUACUGUUGUAGAUCCGAAGCCUGUCAGUCAUAA |
| crRNA-213 | GGGUGGAUAAUUUCUACUGUUGUAGAUCCCGAAGCCUGUCAGUCAUA |

**Table S3** RPA primers designed for the detection of *P. hominis* and *T. foetus.*

| Primer’s name | Primer’s sequence |
| --- | --- |
| F1 | TGTAAACGATGCCGACAGAGGTTTGTCATTT |
| F2 | TGCCGACAGAGGTTTGTCATTTTATAATGGC |
| F3 | GAACTTTTAGAGAAATCATAGTTCTTGGGCT |
| F4 | TAAACGATGCCGACAGAGGTTTGTCATTT |
| F5 | GTAAACGATGCCGACAGAGGTTTGTCATTT |
| F6 | AAACGATGCCGACAGAGGTTTGTCATTT |
| F7 | CGATGCCGACAGAGGTTTGTCATTTTATA |
| F8 | AACGATGCCGACAGAGGTTTGTCATTTTA |
| R1 | CTGAAAGACCCGAAGCCTGTCAGTCATAAAA |
| R2 | AACATCTGGTCCTGGTAAGTTTCCCCGTGTT |
| R3 | GCAATATCCTGAAAGACCCGAAGCCTGTCAG |
| R4 | AAAGCAATATCCTGAAAGACCCGAAGCC |
| R5 | CCCGTGTTGATTCAAATTAAGCCACAGG |
| R6 | GCAATATCCTGAAAGACCCGAAGCCTGT |
| R7 | TGAAAGACCCGAAGCCTGTCAGTCATAA |
| R8 | CAATATCCTGAAAGACCCGAAGCCTGTCA |
| R9 | ACCAACGGCCATGCACCACCAAAAGCAAT |
| R10 | AAGCAATATCCTGAAAGACCCGAAGCCTG |

**Table S4** RPA primers for amplifying *Spo11-1* gene in conjunction with crRNA-412.

| 引物名称  Primer's name | 引物序列  Primer's sequence |
| --- | --- |
| F1 | AGACCGAATGGAAGCAATTCAGATATTAACCCG |
| F2 | ATTCAGATATTAACCCGATAATAGGAACATATG |
| F3 | TCAGCCTTAGACGATCTCCAAGGGTATTTGCAT |
| F4 | TTTGCATCTTTTATCAAAGTACTAGCAGTTGTT |
| F5 | TAACCCGATAATAGGAACATATGTTCTTTATGA |
| F6 | CATCTTTTATCAAAGTACTAGCAGTTGTTGCTT |
| F7 | GGTGAACATGGTTACAAGCGTGGAGTATTTTAC |
| F8 | AACTCTTCGATCAGGTGAACATGGTTACAAGCG |
| R1 | AGCAATGTCCTCAATTATTCTATCAACAGTAC |
| R2 | TCGATCATTCTAGCAATGTCCTCAATTATTCT |
| R3 | ACATGCAACAATTCCCAATGATGTCCTTGGTA |
| R4 | GTCACAAATCCTCTTGGACATGCAACAATTCC |
| R5 | TCATTCTAGCAATGTCCTCAATTATTCTATCAA |
| R6 | TGGTACATCGATCATTCTAGCAATGTCCTCAAT |

**
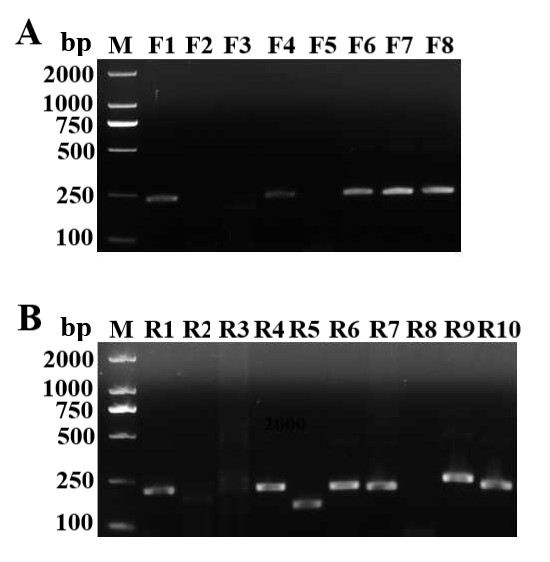
**

**Figure S1** The electrophoresis of RPA primer screen. **A**. The electrophoresis of RPA upstream primer screen; B. The electrophoresis of RPA downstream primer screen. **M**: DNA Maker DL2000.


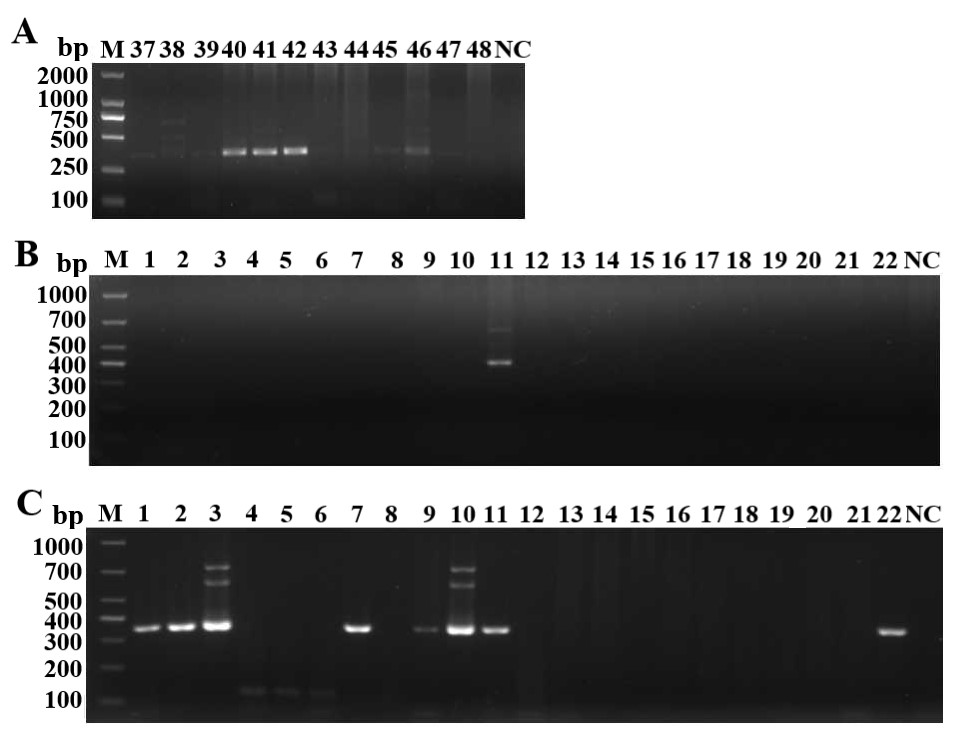


**Figure S2** Electrophoretic analysis of nested PCR amplification for the detection of *P. hominis* and *T. foetus* in fecal samples from dogs and cats **A**. The nested PCR amplification recognizing of *P. hominis* in canine fecal samples; **B**. The nested PCR amplification recognizing of *P. hominis* in feline fecal samples. **C**. The nested PCR amplification recognizing of *T. foetus* in feline fecal samples. **M**: DNA maker DL2000; **NC**: Negative control; **numbers**: canine and feline fecal samples.
